# Excessive infant crying doubles the risk of mood and behavioral problems at age 5: evidence for mediation by maternal characteristics

**DOI:** 10.1007/s00787-016-0888-4

**Published:** 2016-07-15

**Authors:** Laetitia Joanna Clara Antonia Smarius, Thea G. A. Strieder, Eva M. Loomans, Theo A. H. Doreleijers, Tanja G. M. Vrijkotte, Reinoud J. Gemke, Manon van Eijsden

**Affiliations:** 1 Academic Center for Child and Adolescent Psychiatry de Bascule, Meibergdreef 5, 1105AZ Amsterdam, The Netherlands; 2 Department of Epidemiology, Health Promotion and Health Care Innovation Public Health Service Amsterdam, Amsterdam, The Netherlands; 3 Department of Child and Adolescent Psychiatry, VU University Medical Center, Amsterdam, The Netherlands; 4 Department of Social Medicine, Academic Medical Center, University of Amsterdam, Amsterdam, The Netherlands; 5 Department of Pediatrics and EMGO+ Institute for Health and Care Research, VU University Medical Center, Amsterdam, 1007 MB The Netherlands

**Keywords:** Excessive infant crying, Mood problems, Behavioral problems, Maternal burden of infant care, Maternal aggressive behavior

## Abstract

The onset of behavioral problems starts in early life. This study examined whether excessive infant crying (maternal ratings) is a determinant of emotional and behavioral problems at age 5–6 years. In the Amsterdam Born Children and their Development (ABCD) study, a large prospective, observational, population-based multiethnic birth cohort, excessive infant crying (crying for three or more hours per 24 h day over the past week) during the 13th week after birth (range 11–25 weeks, SD 2 weeks), maternal burden of infant care and maternal aggressive behavior (either angry speaking, or physical aggression) was assessed using a questionnaire. Children’s behavioral and emotional problems at the age of 5–6 were assessed by Goodman’s Strengths and Difficulties Questionnaire (SDQ), by the subscale of generalized anxiety of the preschool anxiety scale (PAS), and by the Short Mood and Feelings Questionnaire (SMFQ). Inclusion criterion was singleton birth. Exclusion criteria were preterm born babies or congenital disorders. Among 3389 children, excessive infant crying (*n* = 102) was associated with a twofold increased risk of the overall problem behavior, conduct problems, hyperactivity, and mood problems at the age of 5–6 [ORs between 1.75 (95 % CI 1.09–2.81) and 2.12 (95 % CI 1.30–3.46)]. This association was mediated by maternal burden of infant care (change in odds’ ratio 1–17 %) and maternal aggressive behavior (change in odds’ ratio 4–10 %). There was no effect modification by the child’s gender or maternal parity. Excessive infant crying was not associated with general anxiety problems. Excessive infant crying doubles the risk of behavioral, hyperactivity, and mood problems at the age of 5–6, as reported by their mother. Maternal burden of infant care partially mediates the association between excessive crying and behavioral and mood problems. Special care for mothers with a high burden of care for their excessive crying infant, notwithstanding their own good health, can be a feasible strategy for possible prevention of mood and behavioral problems in their children later in life.

## Introduction

Known risk factors for behavioral problems in childhood include the so-called regulatory problems, such as excessive crying, sleeping, and feeding problems, which occur in 20 % of infants in multiproblem families [1]. Regulatory problems are particularly strong associated with externalizing problems and Attention Deficit Hyperactivity Disorder [2]. In families at risk, excessive crying, whining, and sleeping problems at 4–6 months are associated with decreased social development at 12 months. Feeding problems had no effect on child’s early development [3]. However, these studies provide incomplete information. As far, studies have only examined high-risk populations from high-income countries. Recently, in the Pelotas birth cohort [4], a population of a less high-income country, excessive crying infants were more at risk of behavioral problems in early childhood, compared to other infants. These infants were classified as excessive crying infants when their mother perceived them as crying more than other infants, at the age of 3 months. Unfortunately, no specific information was mentioned on the amount of hours a day, and the infants were crying. Available studies on regulatory problems in infancy and later emotional or behavioral problems did not focus specifically on excessive crying.

Several factors may contribute to, and partly explain, an association between excessive infant crying and later behavioral and emotional problems. During early infancy, the quality of the mother–child dyad can be considered to be a crucial vehicle for child’s healthy mental development. Both early maternal and early paternal reciprocity in infancy are predictive of social competence and lower aggression in preschoolers [5]. The quality of the relationship with the attachment figure is very important for the development of thoughts and feelings [6]. Secure infant-mother attachment at 1 year of age is indeed associated with emotionally matched dialogues between mother and child at the age of 4.5 [7]. One of the most challenging task for parents to manage, however, is prolonged and inconsolable infant crying. In a high-risk group, persistent infant crying was associated with a higher rate of maternal post-partum depressive symptoms, maternal stress, dysfunctional mother–child interactions, perception of the infant as being « difficult » , as well as bonding problems [8, 9]. Indeed, mothers who reported excessive crying were 5.7 times more likely to score high on the parenting stress index (PSI) [10]. Moreover, 5.6 % of the parents of 6 months olds reported having smothered, slapped, or shaken their baby because of its crying [11]. In addition, women with an insecure attachment representation experience more irritation during infant crying and use more excessive force than women with a secure representation [12].

Lasting interactional difficulties might interfere with the healthy development of parent–child and parent–parent dyads [13]. The quality of the mother–child dyad is depending on the amount of stress the mother can manage. Among infants of mothers reporting high prenatal stress, crying appeared inversely associated with maternal levels of self-efficacy [14], suggesting a positive impact of maternal self-efficacy on a healthy mother–child dyad. Avoidant maternal attachment style and post-partum depression are predictors of early childhood developmental problems [15, 16]. Physical abuse is a strong predictor of externalizing behavior in early elementary school aged children [17]. Preschoolers who have been neglected or emotionally abused exhibit a range of serious emotional and behavioral difficulties and adverse mother–child interactions [18]. Maternal high burden of infant care and/or maternal aggressive behavior might influence maternal perception of her infant and, consequently, the quality of their mother–child dyad.

An association between excessive infant crying and emotional or behavioral problems in childhood could also differ between boys and girls or between children with or without older siblings. In newborns admitted to neonatal care, regulatory problems were more strongly associated with deficits in behavior and social skills in boys than in girls [2]. The presence of older siblings is associated with relatively good mental health, compared to having younger siblings [19, 20]. Having no older siblings is a predictor of internalizing problems [21].

The aim of this study was to investigate prospectively the association between excessive infant crying as a single stress regulation indicator and overall behavioral problems, conduct problems, emotional symptoms, hyperactivity/inattention problems, peer relationship problems, pro-social behavior, and mood and general anxiety problems at the age of 5–6 years. Moreover, we examined mediation by maternal burden of infant care and maternal aggressive behavior. Finally, effect modification by child’s gender and maternal parity, as an indirect measure of having older siblings, was tested.

## Methods

### Participants and research design

The study sample is part of a large prospective, observational, population-based multiethnic birth cohort, and the Amsterdam Born Children and their Development (ABCD) study, starting in 2003. Extensive information about cohort and procedures regarding data collection is provided elsewhere [22]. All participating mothers gave their written informed consent. Data were processed anonymously. The approval of the study was obtained from the Central Committee on Research involving Human Subjects in The Netherlands, the Medical Ethical Committees of participating hospitals, and the Registration Committee of the Municipality of Amsterdam.

**Fig. 1:**
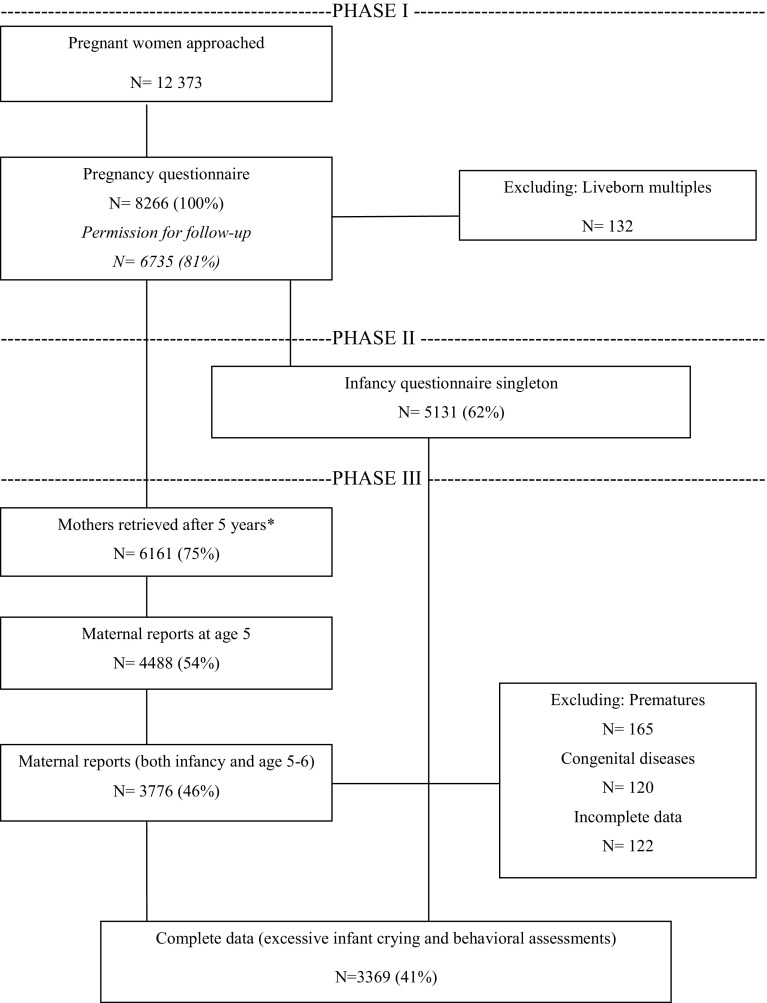
Flowchart of participants included for analysis: *asterisk* attrition at this stage due to withdrawal, infant or maternal death, and unknown address or emigration

The flow chart of participants included is presented in Fig. 1. Between January 2003 and March 2004, all pregnant women living in Amsterdam were asked to participate in the ABCD study during their first prenatal visit to an obstetric care provider (general practitioner, midwife, or gynecologist). Altogether, 12,373 women were approached, which was the total of all new pregnancies in the Amsterdam area during that time period. Of the 12,373 women approached, 8266 women filled out the pregnancy questionnaire [(mean gestational age 15.7 weeks (SD 3.5) (response rate 67 %)]. To investigate the degree of selection bias resulting from non-response, we conducted a non-response analysis by probabilistic medical record linkage with the Netherlands Perinatal Registry. The ABCD non-respondents were significantly younger, more often originating from a non-industrialized country, and more often multiparae. Non-respondents entered antenatal care later were more often under supervision of an obstetrician and had a spontaneous delivery more often. Non-response, however, was not associated with preterm birth or low birth weight. Concluding, anonymised record linkage of cohort study data with national registry data indicated that selective non-response was present in the ABCD study, but selection bias was acceptably low and did not influence the main study questions on adverse pregnancy outcomes [23].

6735 women gave permission for follow-up. Three months after birth, 5131 women filled out the infancy questionnaire. For the questionnaire at the child’s age of 5–6, addresses of 6161 mothers were retrieved. 4488 mothers returned age 5–6 questionnaire. Data on all three measurements (pregnancy, infancy, and early childhood) had to be available to be included in our sample. Children who were twins, born prematurely or had congenital diseases that were excluded.

### Excessive crying in infancy and children’s problem behavior at age 5–6

Excessive infant crying was assessed by an item in the infancy questionnaire which was completed in the 13th week after birth (range 11–25 weeks, SD 2 weeks). Mothers were asked to estimate the number of hours their baby cried on average, over each 24 h period over the past week. Excessive crying was defined as crying for three or more hours per 24 h day on average in the past week according to the mother (best approximation of the Wessel’s criteria) [24].

Children’s behavioral problems and emotional symptoms were reported by their mothers using the Dutch translation of Goodman’s Strengths and Difficulties Questionnaire (SDQ) [25], a short behavioral screening questionnaire suitable for 4–16 year olds. The SDQ consists of 25 items on 5 subscales (conduct problems, emotional symptoms, hyperactivity/inattention problems, peer relationship problems, and pro-social behavior) as well as an overall sum score (total difficulties score). Good reliability and validity of the SDQ have been demonstrated in the Dutch multiethnic population for this age group (age 5–6) (total difficulties score Crohnbach’s alpha 0.77 for parent SDQ, inter-rater correlations 0.21–0.44). The subscales of the SDQ should be interpreted with caution, because the reliability of the SDQ subscales is poorer compared to the total difficulties score, especially in children with a non-Western ethnic background and in 5- to 6-year-old children [26]. Behavioral outcomes were dichotomized into ‘no problem behavior’ and ‘at risk for problem behavior’. Children with a (subscale) score equal to or above the clinically based cutoff of the 80th percentile [27] were considered to be at risk for behavioral, hyperactivity, or emotional problems. Children with a (subscale) score equal to or below the 20th percentile were considered to be at risk for decreased pro-social behavior.

Children’s general anxiety was measured by the generalized anxiety subscale of the validated preschool anxiety scale (PAS) [28], a 28 items questionnaire filled out by the mother. Children who scored equal to or above the 80th percentile on the PAS generalized anxiety subscale were considered to be at risk for generalized anxiety problems. Children’s mood was measured by the validated short mood and feelings questionnaire (SMFQ) reported by mother [29, 30]. Children with a (subscale) score equal to or above the 80th percentile were considered to be at risk for mood problems.

### Potential mediators, moderators, and confounders

Possible mediation by maternal burden of infant care and maternal aggressive behavior was assessed by maternal self-report questionnaire at the infant’s age of 3 months. Maternal burden of infant care was measured using 5 questions (yes or no): ‘all things considered, taking care of my baby is not so hard?; taking care of my baby is quite a burden to me?; the care of my baby takes up so much of my energy that other family members are neglected?; my baby is not easy to take care of?; taking care of my baby is too demanding for me?’ The summed scores were dichotomized, using the 90th percentile as a cutoff. Maternal aggressive behavior (coercive interaction) consisted of four behaviors which were dichotomized [angry speaking (frequency ≤1 or ≥2), cloth on mouth (frequency 0 or ≥1), slapping (frequency 0 or ≥1), and shaking baby (frequency 0 or ≥1)]. These last three behaviors were scored as physical aggression if one out of three was present or not (yes or no). Angry speaking and physical aggression were combined into maternal aggressive behavior (yes or no). Maternal aggressive behavior was considered present if either angry speaking or physical aggression was present. Moderation by parity (1 or ≥2) and gender was tested.

Other potential covariates were selected. The following maternal characteristics during infancy, at the baby’s age of 3 months, were included: age, country of birth (industrialized country or non-industrialized country), cohabitation status (single or with partner), level of education (mean years after primary school), maternal smoking at home (yes or no), and maternal depressive symptoms. Maternal depressive symptoms were measured on a continuous scale using the Center for Epidemiologic Studies Depression Scale (CES-D) [31, 32], as the CES-D does not have an established cutoff for new mothers.

Covariates at age 5 included authoritarian parenting style and maternal stress. Authoritarian parenting style was measured as the 12-items subscale of the short version of the parenting styles and dimensions questionnaire (PSDQ) [33]. The summed scores were dichotomized, using the 90th percentile as a cutoff. Maternal stress at age 5 was measured continuously by the stress severity subscale of the Depression Anxiety Stress Scales DASS 21 [34, 35].

Analyses were conducted using SPSS 19.0 (SPSS inc, Chicago, IL, USA). Descriptive exploratory statistics were used. Statistical differences were tested using the analysis of variance and Chi square. Covariates that were significantly associated with both excessive crying and emotional or behavioral problems were included in a multivariate logistic regression model using a forced–entry method. After initial testing in a crude model, covariates were added to an adjusted, second model. Potential mediation by maternal burden of infant care and maternal aggressive behavior was examined in a third and fourth steps, after an association of these variables with excessive infant crying and with emotional and behavioral problems had been demonstrated. The effect of the mediator was quantified as the percentage of change in odds’ ratio due to adding the mediator to the model, using the formula: [(OR-_adjusted_ _model without mediator_−OR-_adjusted_ _model with mediator_)/(1−OR_-adjusted model without mediator_) × 100 ], provided that the adjusted model showed a significant association [36]. Interaction terms with the child’s gender and maternal parity were added to the crude models to investigate effect modification.

$$ {\text{Mediator effect}} = [({\text{OR}}_{\text{adjusted model without mediator}} - {\text{OR}}_{\text{adjusted model with mediator}} )/(1 - {\text{OR}}_{\text{adjusted model without mediator}} ) \times 100]. $$

## Results

### Subject characteristics

From 3389 included children, complete data on excessive crying and behavioral assessments were available of 3369 children. Mothers who filled out both the infancy questionnaire in ABCD study phase II and reported a rating of their children’s behavior at age 5–6 in phase III (responders, *n* = 3481), differed in key characteristics from mothers who did not report at age 5–6 (non-responders, *n* = 1251). Responders were higher educated (on average one year more), originated more often from industrialized countries (83.8 % vs. 73.9 %, *p* < 0.001), and had less often an excessive crying infant (3.2 % vs. 5.1 %, *p* = 0.002) than non-responders. Responders and non-responders did not differ in maternal burden of infant care and maternal aggressive behavior. The children of responders and non-responders did not differ in birth weight and gestational age.

Compared to other infants, excessive crying infants had a slightly lower birth weight and a slightly younger gestational age. Excessive crying infants more often had a single, lower educated mother, originating from a non-industrialized country, who reported more depression, a higher burden of infant care, and more aggressive behavior and had an authoritarian parenting style. At the age of 5–6, these children had higher SDQ scores and mood and general anxiety scores according to their mothers, compared to non-excessive crying infants (Table 1).

**Table 1:** Demographic characteristics of 3389 women and their children according to excessive crying status

	*N*	% or mean (SD)	Excessive crying *N* = 102 (3.0 %)	Non-excessive crying *N* = 3287 (97.0 %)	*P*
Child characteristics	3389				
Female	1686	49.7 %	54 (52.9 %)	1632 (49.7 %)	0.290
Birth weight, grams	3378	3533 (485)	3364 (529)	3539 (483)	<0.001
Gestational age, wk	3389	39.7 (1.2)	39.4 (1.3)	39.7 (1.2)	0.002
Maternal characteristics					
Primipara	1964	58 %	54 (52.9 %)	1910 (58.1 %)	0.174
Maternal age, years	3389	31.9 (4.4)	29.5 (5.2)	32.0 (4.4)	<0.001
Cohabitancy:living with partner	3076	90.9 %	86 (84.3 %)	2990 (91.1 %)	0.020
Education, years after primary school	3377	10.0 (3.5)	8.1 (4.1)	10.1 (3.4)	<0.001
Ethnic background					<0.001
Industrialized	2883	85.1 %	69 (67.6 %)	2814 (85.6 %)	
Non-industrialized	506	14.9 %	33 (32.4 %)	473 (14.4 %)	
Burden of infant care:high	539	16.0 %	46 (45.1 %)	493 (15.1 %)	<0.001
Depression (Ces-D)	3383	28.4 (6.9)	33.4 (8.0)	28.2 (6.8)	<0.001
Aggressive behavior: yes	373	11.0 %	27 (26.5 %)	346 (10.5 %)	<0.001
Smoking at home: yes	131	3.9 %	7 (6.9 %)	124 (3.8 %)	0.097
Authoritarian parenting style at age 5 (high)	342	11.0 %	19 (22.1 %)	323 (10.7 %)	0.001
Maternal stress at age 5	3076	9.7 (2.8)	10.2 (3.2)	9.6 (2.8)	0.066
Preschooler SDQ scores					
Overall problem behavior	3369	4.9 (3.9)	7.6 (4.8)	4.9 (3.8)	<0.001
Hyperactivity/inattention	3371	2.3 (2.1)	3.3 (2.4)	2.3 (2.1)	<0.001
Emotional symptoms	3371	0.9 (1.2)	1.3 (1.5)	0.9 (1.2)	<0.001
Conduct problems	3369	1.0 (1.2)	1.5 (1.5)	1.0 (1.2)	<0.001
Peer relationship problems	3371	0.7 (1.2)	1.4 (1.8)	0.7 (1.1)	<0.001
Pro-social behavior	3361	8.1 (1.7)	7.6 (1.9)	8.1 (1.7)	0.007
Generalized anxiety P80	3369	853	41 (40.2 %)	812 (24.9 %)	0.001
SMFQ	3357	0.62 (0.7)	0.92 (0.8)	0.62 (0.7)	<0.001

Data are given as percentages or means (±SD)

*SDQ* Goodman’s strengths and difficulties questionnaire, *SMFQ* short mood and feelings questionnaire

### Association between excessive crying in infancy and children’s problem behavior

Excessive crying was associated with a higher risk for hyperactivity/inattention problems, emotional symptoms, conduct problems, peer relationship problems, and overall problem behavior at the age of 5–6, as well as a higher risk for decreased pro-social behavior as reported by the mother (Table 2). Excessive crying was also associated with mood problems as well as generalized anxiety problems at the age of 5–6. After adjusting for confounding factors, an association remained between excessive crying and overall problem behavior [OR 2.12 (1.30–3.46)], conduct problems [OR 1.75 (1.09–2.81)], hyperactivity [OR 1.76 (1.10–2.82)], and mood problems [OR 1.84 (1.14–2.96)] at the age of 5–6. In multivariate analysis, no association remained between excessive infant crying and general anxiety problems.

**Table 2:** Risk of problem behavior in 5-year-old children according to excessive crying status in infancy compared to non-excessive crying (maternal ratings)

	Crude model 1 OR (95 % CI)	Model 2 OR (95 % CI)	Model 3 OR (95 % CI)	Mediating effect of maternal burden of care (%)	Model 4 OR (95 % CI)	Mediating effect of maternal aggressive behavior (%)
Overall problem score P80	3.27 (2.11–5.07)	2.12 (1.30–3.46)	1.98 (1.21–3.24)	12.5 %	1.90 (1.16–3.1 2)	9 %
Conduct problems P80	2.25 (1.46–3.48)	1.75 (1.09–2.81)	1.63 (1.00–2.62)	16 %	1.61 (0.99–2.56)	6 %
Emotional symptoms P80	2.13 (1.37–3.34)	1.54 (0.96–2.47)	1.42 (0.88–2.28)		1.41 (0.88–2.28)	
Hyperactivity P80	2.28 (1.47–3.53)	1.76 (1.10–2.82)	1.75 (1.09–2.82)	1 %	1.72 (1.07–2.78)	4 %
Peer problems P80	2.55 (1.62–4.01)	1.63 (0.79–2.73)	1.61 (0.96–2.70)		1.60 (0.95–2.70)	
Pro-social P20	1.74 (1.08–2.80)	1.45 (0.87–2.42)	1.36 (0.81–2.28)		1.32 (0.79–2.22)	
SMFQ P80	2.62 (1.70–4.05)	1.84 (1.14–2.96)	1.70 (1.05–2.74)	17 %	1.63 (1.01–2.65)	10 %
Generalized anxiety P80	1.87 (1.19–2.92)	1.25 (0.77–2.02)	1.19 (0.74–1.94)		1.17 (0.72–1.90)	

Model 2: adjusted for confounders and moderators (sex, ethnic background, parity, maternal education, maternal depression, smoking at home, and authoritarian parenting style); Model 3: Model 2 additionally adjusted for potential mediator burden of infant care provided that model 2 showed a significant association; Model 4: Model 3 additionally adjusted for potential mediator maternal aggressive behavior provided that model 2 showed a significant association

Evidence was found for mediation by maternal burden of her infant care (change in odds’ ratio by introducing the mediator variable 1–17 %) and by maternal aggressive behavior (change in odds’ ratio 4–10 %). Correlation between the two mediators was low: 0.18 (*p* < 0.001), which substantiates their separate analysis as mediators.

The child’s gender and maternal parity did not moderate the association with excessive crying and behavioral or emotional problems at the age of 5–6 (test for interaction *p* values between 0.330 and 0.911).

## Discussion

Our results show that even in a general population, with a relatively high socio-economic status, excessive infant crying is associated with a twofold increased risk for the overall problem behavior as well as conduct problems, hyperactivity, and mood problems in children at the age of 5–6, as reported by their mothers.

The odds’ ratios we found on behavioral problems are in agreement with results from a meta-analysis by Hemmi on infant regulatory problems and development of the overall behavioral problems between ages 1 and 10 years in multiproblem families [1]. Although excessive crying was less frequently reported by the mothers in our response group, the association of excessive crying with the overall problem behavior at age 5–6 showed an OR of 1.98, which is more robust than the OR of 1.34 found in the Pelotas birth cohort, which is a lower SES population [4]. Therefore, regardless of socio-economic status or known high-risk population or presence of maternal depression, our results suggest that excessive crying in infancy as perceived by their mother is an independent determinant of emotional and behavioral problems at age 5–6.

To the best of our knowledge, we are the first to report that maternal burden of infant care, and to a lesser extent maternal aggressive behavior, partially mediate the association between excessive crying and mood and behavioral problems at the age of 5–6. The findings on maternal aggressive behavior are in accordance with research findings on parental stress and harsh discipline, both of which are determinants of externalizing behaviors at the age of 3. The findings on maternal burden of care are in accordance with research findings on parenting as a determinant of internalizing behaviors at the age of 3 [37]. The mediating role of maternal burden of infant care was smallest in the relationship between excessive infant crying and hyperactivity at the age of 5–6 (1 %). Indeed, it is known that hyperactivity as a symptom in ADHD is explained to a larger extent by genetic blueprint than by environmental factors. Behavioral studies suggest a heritability of approximately 76 % [38].

In earlier research, male as compared to female infants were more vulnerable to high levels of maternal depressive symptoms [39]. However, in our study sample, we found no difference between boys and girls in excessive crying or in maternal burden of infant care (data not shown). We also found no evidence for moderation by the child’s gender or by maternal parity.

### Strengths

This study was conducted in a large, prospective, population-based, multiethnic birth cohort. Assessment of multiple outcomes was possible. The response rate of the study (initial participation, phase I) was 67 %, which is lower than, for example, large population-based pregnancy cohorts in the UK, such as the Southampton Women’s Survey (75 %) [40] and ALSPAC (85 %) [41], but higher than, for example, the Generation R study in Rotterdam, the Netherlands (61 %) [42]. Particularly, in an era where response rates to large epidemiological studies are decreasing [43], our response rate may be considered adequate. Our attrition analysis showed that ABCD study phase III responders did not differ from non-responders in the mediators’ maternal burden of infant care and maternal aggressive behavior.

In the literature, there is no consensus regarding the definition of excessive crying. In this study, excessive crying was defined as crying for three or more hours per 24 h day, on average in the past week, according to the mother, and was assessed during the 13th week after birth. Our definition of excessive crying is more strict than Wessel’s definition (crying > 3 h a day for > 3 days in the preceding week and in the first 6 months of life) [44]; therefore, the number of excessive crying babies is likely not to be overestimated. Although excessive infant crying, feeding, and sleeping problems in the first year of life are highly intertwined, we focused our study specifically on excessive infant crying. We assumed excessive infant crying to be partly an expression of feeding and sleeping problems at the age of 3 months. Because feeding and sleeping problems would possibly precede infant crying at the age of 3 months, and would be part of the causal pathway, correction for feeding and sleeping problems would mean overcorrection of the association. We controlled for a large number of confounding factors that have been demonstrated to be relevant, e.g., ethnic background and authoritarian parenting style, which are associated with greater preschool externalizing problems [45].

### Limitations

In the current study design, maternal perception of the child’s emotion and behavior was measured rather than children’s emotions and behaviors themselves. Importantly, maternal perceptions of her infant do have an effect on maternal sensitivity to their infant, sometimes leading to misattunement between mother and infant: infant negative temperament is positively related to maternal sensitivity when maternal perception of infant soothability is high, but they are inversely related when maternal perception of infant soothability is low [46]. Moreover, it might be argued that the prevalence of children’s problem behavior is overrated by mothers in conditions of maternal stress, resulting in reporting bias. To the contrary, however, in a study on child internalizing behavior in 10–12 year olds, maternal depressive symptoms did not bias maternal reporting on children’s internalizing problems to a serious degree [47]. In our study, maternal stress at the child’s age of 5 did not differ between the excessive criers and the non-excessive criers, suggesting such bias to be minimal.

Another limitation is that paternal report was not collected. Earlier research showed that ratings of excessive crying did not differ between fathers and mothers [48]. Therefore, this is not expected to lessen the validity of our findings. Adjusting for maternal cohabitation/marital status further strengthened the methodological robustness. Data on trauma in early childhood were limited to maternal aggressive behavior in infancy. However, even in the absence of further data on abuse and neglect in early childhood, the available maternal characteristics indicated and mediated an increased risk.

### Implications

Our results suggest that intervention on excessive crying is especially relevant when maternal burden of infant care is high. Maternal burden of care mediates 16 % of the association between excessive crying and conduct problems and 17 % of the association between excessive crying and mood problems at the age of 5–6. As such, we do have a window of opportunity to lighten maternal burden of care to prevent emotional and behavioral problems in their children later in life. Mothers can be offered assistance by easily accessible support services provided by family health visitors to change her perspective of herself and her infant into a healthy mother–child dyad. At the moment, interventions involving mother–infant interaction are focused solely on preterm infants or infants of mothers with a depressive disorder [49, 50].

The results of interventions involving mother–infant interaction are promising, for example, in preterm infants with regulatory problems [51]. Especially in the case of maltreatment, the short-term attachment-based intervention is effective in enhancing parental sensitivity, improving child security, and reducing disorganization for children in the early childhood period [52]. Prevention of emotional and behavioral problems can be expanded to term born babies with excessive crying and non-depressive mothers who experience a high burden of care for their infant notwithstanding their own good health.

## Conclusion

In sum, we found that excessive infant crying as perceived by their mothers, in term born babies, doubles the risk for children’s overall problem behavior, conduct problems, hyperactivity, and mood problems at the age of 5–6. Maternal burden of care for her infant mediates for a small part the association between excessive crying and behavioral and mood problems (12.5–17 %). Mediation by maternal burden of care of the association of excessive crying and hyperactivity is negligible. Evidence was found for partial mediation by maternal aggressive behavior as well (4–10 %). Special care for mothers with a high burden of care for their excessive crying infant notwithstanding their own good health can be a feasible strategy for possible prevention of mood and behavioral problems in their children later in life. Future studies could expand the scope to 10 years of age, to see whether excessive crying infants as perceived by their mother are the children with emotional and behavioral problems as rated by others, for example, their teachers, or by themselves.
